# Role of MicroRNA, LncRNA, and Exosomes in the Progression of Osteoarthritis: A Review of Recent Literature

**DOI:** 10.1111/os.12690

**Published:** 2020-05-20

**Authors:** Fang Xie, Yong‐li Liu, Xiu‐yuan Chen, Qian Li, Jia Zhong, Bin‐yu Dai, Xian‐fang Shao, Guan‐bao Wu

**Affiliations:** ^1^ Affiliated Changde Hospital Hunan University of Traditional Chinese Medicine Changde China; ^2^ Department of Orthopaedics Affiliated Hospital of Hunan Academy of Traditional Chinese Medicine Changsha China

**Keywords:** Exosomes, LncRNA, MicroRNA, Osteoarthritis

## Abstract

Osteoarthritis (OA) is a common clinical degenerative disease characterized by the destruction of articular cartilage, which has an increasing impact on people's lives and social economy. The pathogenesis of OA is complex and unclear, and there is no effective way to block its progress. The study of the pathogenesis of OA is the prerequisite for the early diagnosis and effective treatment of OA. To define the pathogenesis of OA, this review considers the pathological mechanism of OA that involves microRNA, lncRNA, and exosomes. More and more evidence shows that microRNA, lncRNA, and exosomes are closely related to OA. MicroRNA inhibits the target gene by binding to the 3′‐ untranslated region of the targets. LncRNA usually competes with microRNA to regulate the expression level of downstream genes, while exosomes, as a carrier of intercellular information transfer, transmit the biological information of mother cells to target cells, and the effect of exosomes secreted by different cells on OA are different. In this review, we emphasized that different microRNA, lncRNA, and exosomes have different regulatory effects on chondrocyte proliferation and apoptosis, extracellular matrix degradation and inflammation. Besides, we classified and analyzed these molecules according to their effects on the progress of OA. Based on the analysis of the reported literature, this review reveals some pathogenesis of OA, and emphasizes that microRNA, lncRNA, and exosomes have great potential to assist early diagnosis and effective treatment of OA.

## Introduction

Osteoarthritis (OA) is a common degenerative disease related to age, obesity, gender, weight, and trauma^1^. It is characterized by synovial hyperplasia, osteophyte formation, subchondral osteosclerosis, progressive articular cartilage destruction, and cartilage loss caused by the imbalance of extracellular matrix synthesis and catabolism^2^. According to statistics, OA has an impact on the lives of 250 mn people around the world, bringing an annual economic burden of more than US$89.1bn^3^. At present, the treatment strategy for early and middle stage OA is to relieve joint pain and intra‐articular injection, and the treatment strategy for late stage OA is joint replacement surgery. However, although these treatments can alleviate the symptoms of OA and improve the quality of life of patients to a certain extent, they have little effect on blocking the progressive development of OA. The specific pathogenesis of OA is still not clear. The existing evidence shows that the pathogenesis of OA is related to inflammatory factors, abnormal apoptosis of chondrocytes, and degradation of extracellular matrix. During the development of OA, TNF‐a, IL‐1, IL‐6, and other inflammatory factors were abnormally expressed, which led to the increase of chondrocyte apoptosis and the degradation of extracellular matrix^1^, ^4^. At present, there is still a lack of effective means for the early diagnosis and treatment of OA.

**Figure 1:**
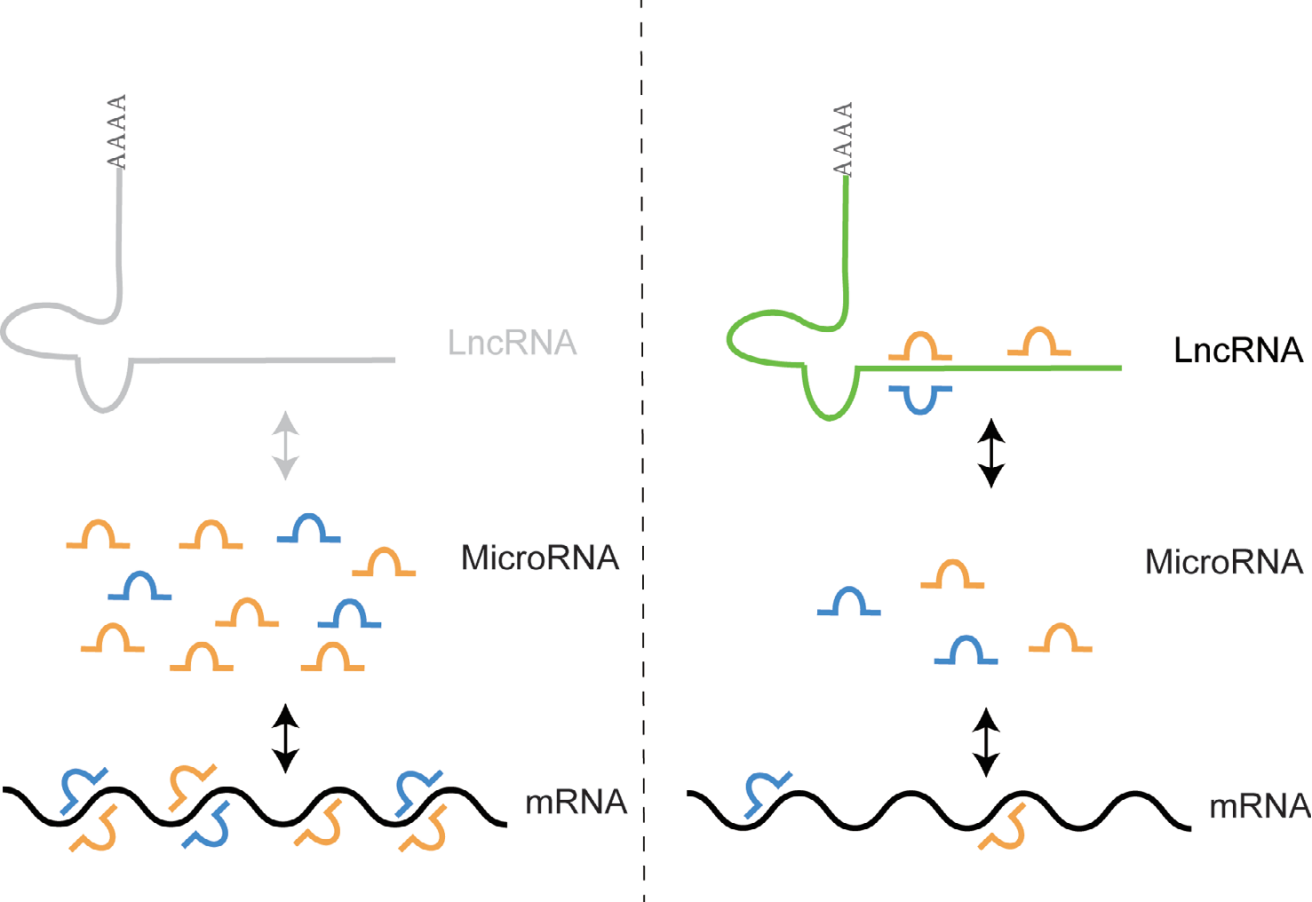
Interaction mechanism of LncRNA, microRNA and mRNA: LncRNA binds and isolates microRNA away from the sites that act on mRNA, thereby reducing the effect of microRNA on mRNA expression.

MicroRNA (miRNA) are a kind of multifunctional non‐coding RNA molecule with 22–25 bases encoded by endogenous genes. MiRNA regulate the stability and translation of mRNA, inhibit splicing and translation, inhibit target gene expression, and regulate downstream pathway by fully complementary binding with the 3′‐ untranslated region (3′‐ UTR) of target mRNA^5^, ^6^, ^7^. Long non‐coding RNA (LncRNA) is a kind of non‐coding RNA with a length of more than 200 bases. It does not encode proteins. It is a regulatory molecule. According to their positions relative to the protein coding genes, it can be divided into five categories: (i) antisense lncRNA; (ii) enhancer lncRNA; (iii) large International non‐coding RNA; (iv) bidirectional lncRNA; and (v) intronic script lncRNA (intron lncRNA)^8^, ^9^. LncRNA binds and isolates microRNA away from the sites that act on mRNA, thereby reducing the effect of microRNA on mRNA expression^10^. Figure 1 illustrates the interaction mechanism of LncRNA, microRNA, and mRNA. Exosomes (Exo) is a kind of vesicle with double plasmalemma structure, which is secreted by cells. It can express CD63, CD9, and other marker proteins on its surface. The membrane contains protein molecules, mRNA, miRNA, lncRNA, and other signal substances, carries the specific cytokines of mother cells, and targets the proximal cells through autocrine and paracrine, or the distal cells through the circulatory system tissue, information exchange between cells^11^, ^12^.

In recent years, with the development of molecular biology technology, the important role of miRNA, lncRNA, and Exo in disease progression has been gradually discovered by researchers. MicroRNA, lncRNA, and Exo are also expected to be an important way to explain the pathogenesis, early diagnosis, and treatment of OA. The purpose of this review is to summarize the key role of microRNA, lncRNA, and Exo in the development of OA. All the information is extracted from the high‐quality literature retrieved in the PubMed database.

## Methods

**Figure 2:**
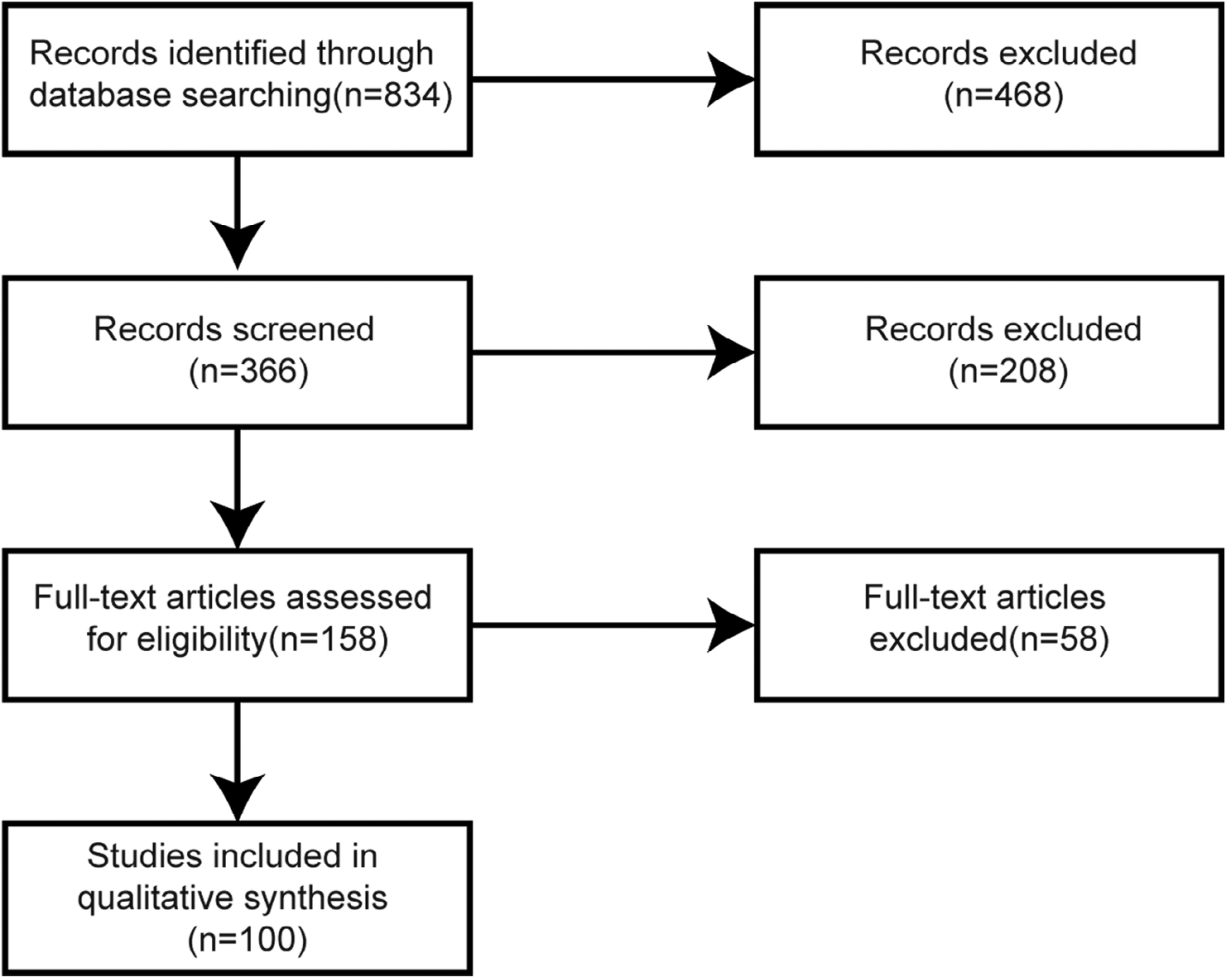
The flow chart of literature search and screening.

With the help of the library platform of Hunan University of Traditional Chinese Medicine, PubMed database was searched. The literature from 2010 to 2019 were searched with the keywords “osteoarthritis,” “microRNA,” “lncRNA,” “exosomes,” and the language was English. Figure 2 illustrates the flow chart of searched results.

Eight hundred and thirty‐four related citations were obtained by literature search. Three hundred and sixty‐six related citations were obtained by taking experimental studies related to the pathological process of OA as the selection criteria and deleting the duplicate part. The two authors independently screened the titles and abstracts of each article, excluding the comprehensive research, clinical research, meeting report, and case report. The screening process was completed by the Rayyan QCRI software, and the dispute part was solved by the third author independently. A total of 158 documents have been full‐text reviewed. Through the full‐text review, there are 100 documents in line with our selection criteria, including 35 documents related to microRNA and OA, 54 documents related to lncRNA and OA, and 11 documents related to exosomes and OA. A descriptive analysis was performed on included studies.

## Results

### *MicroRNA*

In the pathogenesis of OA, miRNA has the biological functions of regulating chondrocyte apoptosis and proliferation, extracellular matrix metabolism, inflammatory response, and so on^13^, ^14^, ^15^, ^16^. The keywords of miRNA and osteoarthritis were searched in the PubMed database, 39 of which were research literature on molecular mechanism. It was found that there were 16 kinds of miRNA inhibiting OA process and 14 kinds of miRNA promoting OA process (Table 1).

**Table 1:** classification of miRNA in OA process

Inhibiting OA process (16 kinds)	Promoting OA process(14 kinds)
miR‐132^17^, ^18^, miR‐107^19^, miR‐149‐5p^20^, miR‐93‐5p^21^, miR‐335‐5p^22^, miR‐4784^23^, miR‐106a‐5p^24^, miR‐145^25^, miR‐140^26^, ^27^, miR‐221^28^, miR‐381^29^, miR‐105^30^, miR‐210^31^, miR‐29a^27^, miR‐488^32^, miR‐125b^33^, miR‐101^34^	miR‐146b^35^, ^36^, miR‐34a^37^, ^38^, miR‐181a^37^, ^39^, ^40^, miR‐582‐5p^41^, miR‐324‐5p^42^, miR‐21‐5p^43^, miR‐483‐5p^44^, ^45^, miR‐384‐5p^46^, miR‐155^39^, miR‐98^47^, miR‐127‐5p^48^, miR‐16‐5p^49^, miR‐101^50^, miR‐146a^51^

Apoptosis is a form of programmed cell death, which involves a series of gene activation, expression, and regulation^52^. Out of control apoptosis can cause cancer, autoimmune diseases, and degenerative diseases. Over apoptosis of chondrocytes is the main pathological manifestation of OA^53^. MiRNA promote or inhibit chondrocyte apoptosis by regulating the expression of key molecules involved in apoptosis signaling pathways. MiR‐34a and miR‐108a have a synergistic relationship in chondrocytes, which can jointly promote the activity of p50 NF‐ κB, reduce the expression of Bcl‐2, and promote chondrocyte apoptosis^37^; in addition, miR‐98 can also inhibit the transcription of Bcl‐2 and the apoptosis of chondrocytes^47^. According to the research of Zhao *et al*.^40^, miR‐181a can inhibit the expression of Glycerol‐3‐Phosphate Dehydrogenase 1‐Like Protein (GPD1L) and accelerate the apoptosis of chondrocytes by binding with the 3′‐ UTR end of GPD1L. In addition, miR‐181a may have a cooperative relationship with miR‐155^39^. There are other targets in miR‐34a, and Yang *et al*.^38^ have proved that miR‐34a targets cysteine‐rich angiogenic inductor 61 (CYR61), and inhibiting the expression of miR‐34a can promote the proliferation of chondrocytes. MiR‐146b can inhibit the expression of alpha‐2‐macroglobulin (A2M), improve the activity of proteolytic enzyme, promote cell apoptosis, and accelerate the development of OA^35^. MiR‐384‐5p inhibit cell proliferation and induce apoptosis by targeting Sox9^46^. MiR‐127‐5p targeting combined with OPN inhibit chondrocyte proliferation^48^. Li *et al*.^31^ showed that miR‐210 inhibit the expression of HIF‐3 α and promoted the proliferation of chondrocytes. MiR‐146a target Smad4 to promote chondrocyte apoptosis^51^. MiR‐29a can upregulate the expression of type II collagen, reduce the expression of MMP13, resist the apoptosis of chondrocytes, and delay the development of OA^27^.

The extracellular matrix (ECM) of articular cartilage is mainly composed of proteoglycan and type II collagen, including a small number of chondrocytes. ECM contains a large number of signal molecules, which actively participate in the control of cell growth, polarity, shape, migration, and metabolism. The disruption of ECM catabolic balance will lead to the occurrence of OA^54^. Wang *et al*.^45^ found that miR‐483‐5p directly target Matn3 and TIMP2 to promote the degradation of ECM, chondrocyte hypertrophy, and cartilage angiogenesis, and accelerate the process of OA. Zheng *et al*.^28^ confirmed that miR‐221 was down regulate in OA, and miR‐221 could inhibit the degradation of cartilage extracellular matrix through SDF1 / CXCR4 signaling pathway. MiR‐145 inhibit the phosphorylation of MMK4 and alleviate the degradation of cartilage extracellular matrix caused by TNF ‐ α stimulation^25^. Liu *et al*.^23^ found that the expression of miR‐4784 is low in the early stage of OA, overexpression of miR‐4784 can increase the expression of type II collagen in cartilage extracellular matrix, decrease the expression of MMP3, and inhibit the degradation of extracellular matrix. Li *et al*.^49^ found that the expression of miR‐16‐5p in OA tissue is higher than that in normal tissue and further experiments confirm that miR‐16‐5p can target Smad3 to promote the degradation of cartilage extracellular matrix and promote the development of OA. In the experiments of Dai *et al*.^50^, they found that silencing miR‐101 can increase the expression of Sox9 and type II collagen and proteoglycan, thus inhibiting the degradation of cartilage extracellular matrix. However, Gao *et al*.^34^ found that the expression of miR‐101 increases while that of Sox9 and Runx2 decreases.

### *Long Non‐Coding* *RNA*

The pathogenesis of OA is still unclear, but a large amount of evidence shows that the interaction between lncRNA and miRNA plays an important role in the development of OA. LncRNA can competitively bind miRNA, act as competitive endogenous RNA (CeRNA), reduce the combination of miRNA and downstream genes, and increase the transcription and expression of downstream genes^55^, ^56^. LncRNA has become an early diagnosis and effective treatment target of OA. In the PubMed database, 45 relevant pieces of literature were searched with the keywords of lncRNA and osteoarthritis, and 34 kinds of lncRNA were involved in these studies, including 17 kinds of lncRNA molecules inhibiting the development of OA and 15 kinds of lncRNA molecules promoting the development of OA (Table 2).

**Table 2:** classification of lncRNA in OA process

	LncRNA	Targets	Cell process
Inhibiting OA process	FOXD2‐AS1^57^, ^58^ ANCR^59^ DILC^60^ MIR4435‐2HG^61^ SNHG1^62^ SNHG5^63^ HULC^64^ PACER^65^ MEG3^66^, ^67^ LINC00341^68^ ATB^69^ PMS2L2^70^ MALAT1^71^ ROR^72^ ZFAS1^73^ GACAT3^74^ UFC1^75^	MiR‐27a/TLR4^57^ MiR‐206/CCND1^58^ MiR‐16‐5P MiR‐26a/Sox2 MiR‐101 MiR‐93/TGFBR2^66^ MiR‐16/SAMD7^67^ MiR‐141/YAF2 MiR‐223 MiR‐203 MiR‐150‐5p/AKT3 MiR‐34a	Chondrocyte proliferation ( + ) Chondrocyte proliferation ( + ) Chondrocyte proliferation ( + ) Inflammation(‐) Chondrocyte proliferation ( + ) Inflammation (‐) Chondrocyte proliferation ( + ) Inflammation (‐) Chondrocyte apoptosis (‐) Degradation of extracellular matrix (‐) Chondrocyte proliferation ( + ) Chondrocyte apoptosis (‐) Inflammation (‐) Inflammation (‐) Chondrocyte proliferation ( + ) Chondrocyte apoptosis (‐) Chondrocyte proliferation ( + ) Chondrocyte proliferation ( + ) Chondrocyte apoptosis (‐)
Promoting OA process	MIAI^17^ DANCR^76^, ^77^ TM1P3^78^ CTD‐2574D22.4^79^ TNFSF10^80^ LOC101928134^81^ CASA2^82^ CHRF^83^ Nespas^84^ H19^85^ THRIL^86^ TUG^87^ P21^88^ CIR^89^, ^90^, ^91^ PVT1^92^, ^93^ XIST^94^ MBNL1‐AS1^95^ HOTAIR^96^ FAS‐AS1^97^ MSR^98^ PCGEM1^99^	MiR‐132 MiR‐216a/JAK2^76^ MiR‐577/Sphk2^77^ MiR‐22/MMP13 MiR‐376/FGFR1 TL‐17 MiR‐146a/JAK1/STAT3 MiR‐130a MiR‐125b MiR‐195/MMP‐13 MiR‐130b/PTEN/AKT MiR‐130a/Bim^89^ MiR‐27b^91^ MiR‐149^92^ MiR‐488^93^ MiR‐211/CXR4/MAPK MiR‐124^96^ MiR‐17‐5p/FUT2/β‐catenin^96^ MiR‐152 MiR‐770	Chondrocyte proliferation (‐) OA Chondrocyte proliferation and inflammation ( + ) OA Chondrocyte proliferation ( + ) Degradation of extracellular matrix ( + ) Chondrocyte apoptosis and inflammation ( + ) OA Chondrocyte proliferation and inflammation ( + ) Chondrocyte apoptosis ( + ) Chondrocyte apoptosis ( + ) Inflammation ( + ) Chondrocyte apoptosis ( + ) Chondrocyte apoptosis ( + ) Inflammation ( + ) Degradation of extracellular matrix ( + ) Chondrocyte apoptosis ( + ) Chondrocyte apoptosis ( + ) Chondrocyte autophagy ( + )^90^ Degradation of extracellular matrix ( + ) Degradation of extracellular matrix ( + ) Chondrocyte apoptosis ( + ) Chondrocyte apoptosis ( + ) Chondrocyte apoptosis ( + ) Inflammation ( + ) Degradation of extracellular matrix ( + ) Degradation of extracellular matrix ( + ) Degradation of extracellular matrix ( + ) Chondrocyte apoptosis ( + )

The combination of lncRNA and miRNA interferes with the inhibition of miRNAs on the expression of downstream target genes. That is to say, the expression of lncRNA is negatively correlated with the corresponding miRNAs and positively correlated with the expression of downstream target genes. Competitive binding of mRNA to miRNA is the main way for lncRNA to regulate biological functions. In the known research, it has been confirmed that some lncRNA have multi‐target and multi‐level regulatory effect. The study of Wang *et al*.^57^ confirmed that lncRNA FOXD2‐AS1 was low expression in OA patients, further experiments show that lncRNA FOXD2‐AS1 promote chondrocyte proliferation and inhibit the development of OA through miR‐27a / TLR4 axis. Cao *et al*.^58^ confirm that lncRNA FOXD2‐AS1 inhibit the expression of miR‐206 and upregulate the expression of CCND1, through miR‐206 / CCND1 axis, and it promote the survival and development of chondrocytes and hinder the process of OA, besides, lncRNA DANCR promote the proliferation of OA chondrocytes through miR‐216a / JAK2 axis 76 and miR‐577 / Sphk273 axis. LncRNA CIR can promote apoptosis through miR‐130a / Bim axis^89^, and inhibit the expression of miR‐27b^91^ to promote the degradation of extracellular matrix. LncRNA PVT1 target regulation of miR‐149^92^ and miR‐488^93^ expression promotes apoptosis, extracellular matrix degradation, and inflammatory response. LncRNA HOTAIR inhibit miR‐124^96^ expression or promote inflammatory response and apoptosis through miR‐17‐5p / FUT2 /β‐ catenin axis^96^.

Jiang *et al*.^65^ found that the expression of lncRNA PACER is low in OA and inhibited cell apoptosis. The negative correlation between the expression of PACER and lncRNA HOTAIR suggested that there is also a mechanism of mutual regulation between lncRNA. There are also some lncRNA that have been proved to be related to OA, but the specific regulatory mechanism is not clear. Li *et al*.^59^ found that lncRNA ANCR can promote chondrocyte proliferation in OA patients, and its expression was negatively correlated with TGF ‐β1. Huang *et al*.^60^ alleviate the inflammatory response in OA by inhibiting the expression of IL‐6. Yang *et al*.^81^ found that when the expression of lncRNA LOC101928134 is downregulated, the expression of IFN1 increase and activate the downstream JAK / STAT signal pathway, inhibiting the proliferation of synovium and cartilage destruction, but the specific regulatory mechanism of LOC101928134 is not clear. In addition, lncRNA ZFAS1 can inhibit the activation of Wnt3a pathway and promote the proliferation and migration of chondrocytes^73^. The expression of GACAT3 is negatively correlated with IL‐6, inhibits the activity of IL‐6 / STAT pathway, and promotes chondrocyte proliferation^74^. The study of Park *et al*.^84^ found that the overexpression of lncRNA Nespas can inhibit the expression of miR‐291a‐3p, miR‐196a‐5p, miR‐23a‐3p, miR‐24‐3p, and Let‐7a‐5p. After analysis, these miRNAs target ACSL6, but the transcriptional binding of these miRNAs with lncRNA Nespas and ACSL6 needs further experimental verification.

Interestingly, Zhang *et al*.^76^ found that the expression of lncRNA DANCR increases in OA patients, DANCR promotes the proliferation and inflammatory response of OA chondrocytes, inhibits apoptosis, and promotes the progression of OA. Fan *et al*.^77^ also found that the expression of DANCR increases in OA patients and promotes the proliferation of OA chondrocytes through miR‐577 / Sphk2 axis. The study of Huang *et al*.^80^ confirmed that lncRNA TNFSF10 promotes OA chondrocyte proliferation, inhibits apoptosis, and promotes inflammatory response through miR‐376 / FGFR1 axis. These experiments prove that OA chondrocytes are relate to inflammatory response, and promoting the proliferation of OA chondrocytes can accelerate the development of OA. In other words, the proliferation and inflammatory response of degenerated chondrocytes can accelerate the degeneration of cartilage^100^. OA chondrocytes have different biological functions from normal chondrocytes.

### *Exosomes*

The biological characteristics of Exo, which is not secreted by cells, are different, and its effect on OA is also different. Domenis *et al*.^101^ extracted and identified synovial‐fluid‐derived exosomes of patients with osteoarthritis, and found that SF‐Exo can promote inflammatory response. The Exo secreted by IL‐1β stimulate synovial fibroblasts can increase the expression of MMP‐13 and ADAMTS5, decrease the expression of COL2A1 and ACAN, promote the degradation of cartilage extracellular matrix, and accelerate the development of OA^102^. Because Exo can carry the biological information of mother cells, more research regards Exo as a strategy of treatment. The study of Qi *et al*.^103^ confirms that Exo secrete by mesenchymal stem cells can promote Akt phosphorylation by inhibiting p38 and ERK phosphorylation, and inhibit chondrocyte apoptosis cause by mitochondrial dysfunction. Bone‐marrow‐mesenchymal‐stem‐cells Exo^104^, embryonic‐mesenchymal‐stem‐cells Exo^105^, and adipose‐mesenchymal‐stem‐cells Exo^106^ can promote the expression of type II collagen and proteoglycan, inhibit the expression of MMP‐13, ADAMTS5 and proinflammatory factors, and maintain the balance of cartilage extracellular matrix. Exosomes can also regulate the biological functions of target cells by carrying miRNA and lncRNA. Wu *et al*.^107^ found that miR‐100‐5p was abundant in the Exo of subpatellar fat pad mesenchymal stem cells, and the activity of mTOP autophagy pathway in chondrocytes is inhibited by miR‐100‐5p to adjust the gait of OA in a rat model. Sun *et al*.^108^ confirmed that Exo can promote chondrocyte proliferation and inhibit MMP‐13 expression through miR‐302c. The study of Mao *et al*.^109^ confirmed that the expression of miR‐92a in Exo of OA chondrocytes was lower than that of normal chondrocytes and further experiments show that human mesenchymal stem cells inhibit the expression of Wnt5a through miR‐92a and alleviate the degeneration of articular cartilage. The Exo secrete by miR‐140‐5p overexpress synovial stem cells can promote the proliferation and migration of chondrocytes^110^. Liu *et al*.^111^ confirmed that human mesenchymal stem cells could inhibit the apoptosis of chondrocytes induced by IL‐1β and promote cartilage repair through lncRNA KLF3‐AS1.

### *Conclusions*

**Figure 3:**
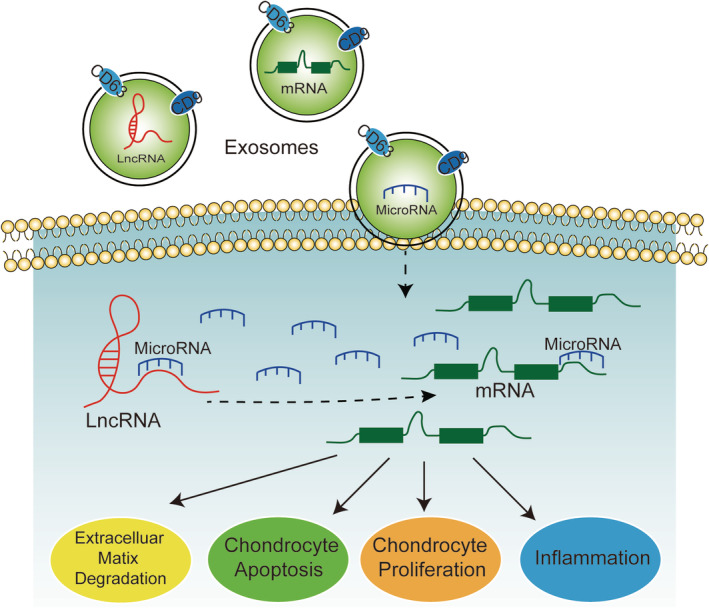
MicroRNA, IncRNA and Exo intervence the pathological process of OA through chondrocyte proliferation, chondrocyte apoptosis, extracellulsr matrix degradation and inflammation.

MicroRNA, lncRNA, and Exo have strong regulatory effects on the pathological process of OA, so they are expected to be the targets of early diagnosis and treatment of OA. Figure 3 illustrates the mechanism of the study. The interaction mechanism of these three factors may be as follows: Exo secreted by other cells contains different lncRNA, microRNA, or mRNA; when Exo contacts the cell membrane, these substances are transferred to the target cell; in the cell, microRNA can bind mRNA to inhibit its expression. However, lncRNA can competitively bind microRNA to alleviate the inhibition of microRNA on the expression of downstream genes. All in all, their regulatory role in cells is ultimately achieved by influencing mRNA expression downstream. From the current literature reports, the pathological research of OA involves microRNA, lncRNA, and Exo, which are basically *in vitro* experiments or animal model experiments. With the progress of molecular biotechnology in the later research, it is hoped that more clinical experimental reports will be published. In addition, due to the diversity of biological functions of microRNA and lncRNA and the characteristics of Exo information transmission media, research into the fusion of these three factors may effectively promote the research process of the pathological mechanism of OA.

### *Authorship Declaration*

All authors listed meet the authorship criteria according to the latest guidelines of the International Committee of Medical Journal Editors, and all authors are in agreement with the manuscript.
